# Mesoporous metallic rhodium nanoparticles

**DOI:** 10.1038/ncomms15581

**Published:** 2017-05-19

**Authors:** Bo Jiang, Cuiling Li, Ömer Dag, Hideki Abe, Toshiaki Takei, Tsubasa Imai, Md. Shahriar A. Hossain, Md. Tofazzal Islam, Kathleen Wood, Joel Henzie, Yusuke Yamauchi

**Affiliations:** 1 International Center for Materials Nanoarchitectonics (MANA), National Institute for Materials Science (NIMS), 1-1 Namiki, Tsukuba, Ibaraki 305-0044, Japan; 2 Faculty of Science and Engineering, Waseda University, 3-4-1 Okubo, Shinjuku, Tokyo 169-8555, Japan; 3 Department of Chemistry, Bilkent University, 06800 Ankara, Turkey; 4 Australian Institute for Innovative Materials (AIIM), University of Wollongong (UOW), Squires Way, North Wollongong, New South Wales 2500, Australia; 5 Department of Biotechnology, Bangabandhu Sheikh Mujibur Rahman Agricultural University, Gazipur 1706, Bangladesh; 6 Australian Nuclear Science and Technology Organisation (ANSTO), New Illawarra Rd, Lucas Heights, New South Wales 2234, Australia

## Abstract

Mesoporous noble metals are an emerging class of cutting-edge nanostructured catalysts due to their abundant exposed active sites and highly accessible surfaces. Although various noble metal (e.g. Pt, Pd and Au) structures have been synthesized by hard- and soft-templating methods, mesoporous rhodium (Rh) nanoparticles have never been generated via chemical reduction, in part due to the relatively high surface energy of rhodium (Rh) metal. Here we describe a simple, scalable route to generate mesoporous Rh by chemical reduction on polymeric micelle templates [poly(ethylene oxide)-*b*-poly(methyl methacrylate) (PEO-*b*-PMMA)]. The mesoporous Rh nanoparticles exhibited a ∼2.6 times enhancement for the electrocatalytic oxidation of methanol compared to commercially available Rh catalyst. Surprisingly, the high surface area mesoporous structure of the Rh catalyst was thermally stable up to 400 °C. The combination of high surface area and thermal stability also enables superior catalytic activity for the remediation of nitric oxide (NO) in lean-burn exhaust containing high concentrations of O_2_.

## Abstract (web summary)

Mesoporous noble metal nanostructures offer great promise in catalytic applications. Here, Yamauchi and co-workers synthesize mesoporous rhodium nanoparticles using polymeric micelle templates, and report appreciable activities for methanol oxidation and NO remediation.

The creation of well-controlled nanomaterial architectures is critical for more efficient utilization of rare metals and also enables unique chemical and physical properties that are not evident in bulk^1^,^2^,^3^. Making mesoporous frameworks composed of carbon^4^,^5^,^6^,^7^, metal oxides^8^,^9^, silica^10^,^11^,^12^,^13^, polymers^14^,^15^,^16^ and metals^17^,^18^,^19^ has led to enhanced performance in catalysis^20^,^21^, energy conversion and storage^22^,^23^, surface-enhanced Raman spectroscopy (SERS)^24^,^25^ and chemical/biochemical sensing^26^. In particular, the application of mesoporous noble metals in the field of heterogeneous catalysis has distinct and numerous advantages in terms of performance. These mesoporous metal architectures expose abundant catalytically active sites due to their high specific surface areas and accessible porous structures. The inherent conductivity of the metal maximizes the rate of electron transfer inside a porous network, helping to facilitate mass transfer throughout the entire structure^27^,^28^. Growing metals inside sacrificial hard-templates (for example, mesoporous silica) has mostly been used to synthesize mesoporous metal structures^29^,^30^. Recently, lyotropic liquid crystals (LLC) composed of surfactants or block polymers were used as soft templates to grow mesoporous metals (e.g. Pt, Pd, Ni, Rh and Sn)^1^,^31^,^32^,^33^. Yet the flexibility of the LLC approach is limited due to the high viscosity of the solvent system, and there have been few reports describing the successful synthesis of mesoporous rhodium (Rh) catalysts thus far. Simplifying the synthesis of mesoporous Rh would expand the materials palette for mesoporous noble metal chemistry and enable more efficient utilization of rare metals in catalysis.

Nanostructured Rh is ubiquitous in heterogeneous catalysis, playing a huge role in the refinement of petroleum, the production of fine chemicals and energy generation via fuel cell technology^34^,^35^,^36^,^37^. Increasing surface area has been a common strategy to improve the performance of catalysts^38^. Advances in synthetic colloidal methods, combined with access to advanced structural characterization tools, has enabled the deliberate engineering of surface atoms, including their degree of coordination, and the presence of structural defects^39^,^40^. Making nano-sized Rh materials should similarly enable a higher level of control over surface structure. For example, Yu *et al*. has shown that the convex Rh nanocrystals bound by {830} facets exhibit higher activity for ethanol electro-oxidation than irregular Rh nanoparticles, as well as commercial Rh black^41^. Huang and co-authors pointed out that wavy Rh nanowires have numerous structural defects/grain boundaries that are responsible for high catalytic activity^42^. Additionally, two-dimensional (2D) Rh nanosheets with exposed Rh atoms in ultrathin layered nanostructures perform well in hydrogenation reactions^43^. Although the precise nature of the Rh active site is not well understood, the impact of crystallographic features (for example, atomic steps, corners and defects) induced by well-designed nanostructures can boost performance in numerous catalytic reactions^41^,^42^,^43^,^44^. Therefore, the synthesis of mesoporous Rh structures should be another effective way to further increase surface area and catalytic active sites while pushing the limits of material utilization beyond what can be achieved with existing Rh-based nanostructures (that is, nanoparticles, nanowires and so on).

The surface energy of Rh is notably larger than other noble metals including Pt, Au and Pd^45^. Thus generating high surface area Rh nanostructures at mild conditions is extremely challenging synthetically because the equilibrium shape of Rh and other FCC metals tends to minimize surface area and is dominated by low index facets^46^. Therefore, a successful synthesis of mesoporous Rh will likely need to be controlled by a kinetic mechanism. Here we describe the synthesis of a nanostructured Rh catalyst and examine how the mesoporous structure impacts its catalytic properties. The synthesis of Rh mesoporous nanoparticles is realized using chemical reduction of a Rh precursor salt on sacrificial polymeric micelle templates. Construction of accessible 3D mesoporous networks in Rh metal maximizes the material utilization of Rh and simultaneously enables the creation of numerous active sites for enhanced catalytic activity.

## Results

### Synthesis of mesoporous Rh nanoparticles

**Figure 1:**
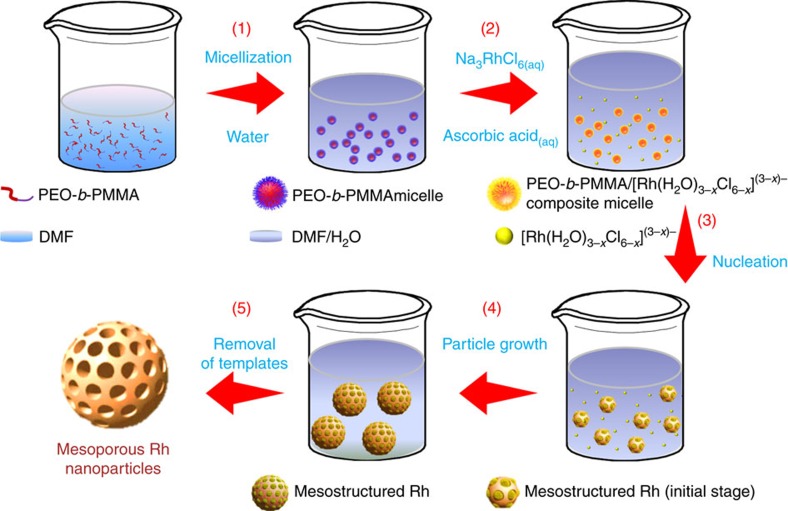
An illustration describing the formation mechanism for mesoporous Rh nanostructures. Mesoporous Rh nanostructures form via chemical reduction on self-assemble polymeric PEO-*b*-PMMA micelle templates. The PEO-*b*-PMMA micelles function as a soft template, Na_3_RhCl_6_ as the Rh precursor, AA as the reducing agent, and DMF/H_2_O as the mixed solvent, respectively. The synthetic process has five steps; (1) addition of water causes the PEO-*b*-PMMA to self-assemble into spherical micelles with a PMMA core and a PEO shell, (2) Na_3_RhCl_6_ source is dissolved into Na^+^ and [Rh(H_2_O)_3–*x*_Cl_6–*x*_]^(3–*x*)−^ and then the aqua-complexes interact with the PEO moieties via hydrogen bonding between the PEO and aqua-complexes (that is, the formation of PEO-*b*-PMMA/[Rh(H_2_O)_3–*x*_Cl_6–*x*_]^(3–*x*)−^ composite micelles), (3) the Rh species are reduced to form solid Rh nuclei, (4) which coalesce and further grow into mesoporous Rh nanostructures over and (5) the templates are removed by a solvent extraction method.

A schematic diagram illustrates the wet chemical synthesis of mesoporous Rh nanoparticles (Fig. 1). A block polymer poly(ethylene oxide)-*b*-poly(methyl methacrylate) (PEO_10500_-*b*-PMMA_18000_) is used as a template, while water and N,N-dimethylformamide (DMF) are used as the mixed solvent, and Na_3_RhCl_6_ and ascorbic acid (AA) were selected as metal precursor and reducing agent, respectively. After chemical reduction, the mesoporous Rh nanoparticles were successfully generated via assembly of polymeric micelles (see Methods). After consecutive washing/centrifugation cycles with an appropriate solvent, the product had a nearly clean surface (Supplementary Fig. 1).

**Figure 2:**
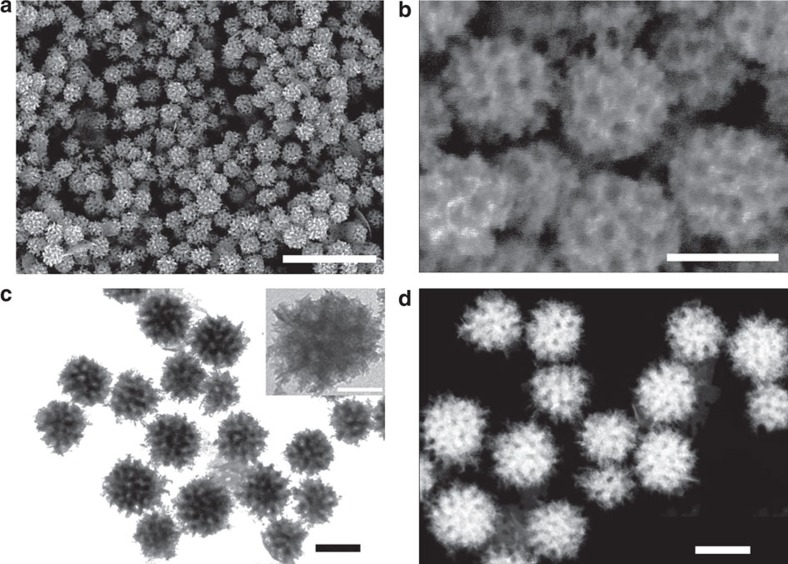
Structural characterization of mesoporous Rh nanoparticles. (**a**) Low-magnification SEM micrograph (Scale bar, 500 nm), (**b**) high-magnification SEM micrograph (Scale bar, 100 nm), (**c**) TEM micrograph (Scale bar, 100 nm) and (**d**) HAADF-STEM micrograph (Scale bar, 100 nm) of mesoporous Rh nanoparticles. The inset in **c** is a high-magnification TEM micrograph of a single mesoporous Rh nanoparticle (Scale bar, 50 nm).

The shape and mesoporous structure of the sample were initially characterized by scanning electron microscopy (SEM) and transmission electron microscopy (TEM) (Fig. 2). According to SEM micrographs, the nanoparticles are highly dispersed with uniformity in both shape and size (Fig. 2a). The average diameter of the nanoparticles was estimated to be ∼100 nm by analysing >200 nanoparticles (Supplementary Fig. 2). SEM shows well-defined mesostructures with large-sized pores distributed throughout the entire outer surface of the nanoparticles (Fig. 2b). Closer inspection using high-angle annular dark-field scanning TEM (HAADF-TEM) shows a pattern of contrast that indicates the mesopores are distributed throughout the interior of the nanoparticles (Fig. 2d). The selected area electron diffraction (SAED) patterns of single particles show concentric rings of spots, indicating the particles are polycrystalline (Supplementary Fig. 3a,b). But closer examination with high-resolution TEM (HRTEM) shows that the local structure around the pore walls is highly crystalline, with 0.20 nm lattice fringes that correspond to the (111) plane of the face-centered cubic (fcc) Rh crystal (Supplementary Fig. 3c,d). Surprisingly, some atomic steps and kink sites typically associated with unsaturated coordination atoms could be observed on the branched structure. Presence of these structures is a good initial sign that these materials will have excellent performance in catalysis.

Low-angle X-ray diffraction measurements were used here to determine the periodicity (that is, pore-to-pore distance) of the final mesoporous structure. A single peak located at 0.43° was observed in the X-ray diffraction pattern, showing the pores are uniform in size and closely packed (Supplementary Fig. 4a). On the basis of centre of the peak, the calculated pore-to-pore distance was ∼21 nm. Considering the pore size (∼11 nm via SEM, Fig. 2b), the thickness of the pore wall was estimated to be ∼10 nm. The wide-angle X-ray diffraction pattern of the sample displays five characteristic diffraction peaks of the Rh (111), (200), (220), (311) and (222), respectively (Supplementary Fig. 4b). No other impurities or rhodium oxide diffraction peaks were detected in the X-ray diffraction patterns, suggesting the particle is purely metallic. The average grain size is ∼4.2 nm according to the Scherrer equation, which we assume is roughly the average size of the sub-particles within the mesoporous networks. The electronic state of Rh surface is also confirmed by X-ray photoelectron spectroscopy (XPS) analysis (Supplementary Fig. 1b). Nitrogen (N_2_) adsorption-desorption isotherms demonstrate the sample has a large surface area of *ca.* 50 m^2^ g^−1^ (Supplementary Fig. 4c). The surface area value is within a reasonable range for mesoporous materials with large mesopores (>10 nm), as shown in Supplementary Table 1. The pore size distribution diagram (Supplementary Fig. 4d) clearly shows the presence of mesopores (around 9 nm) originating from the removal of polymeric micelles.

**Figure 3:**
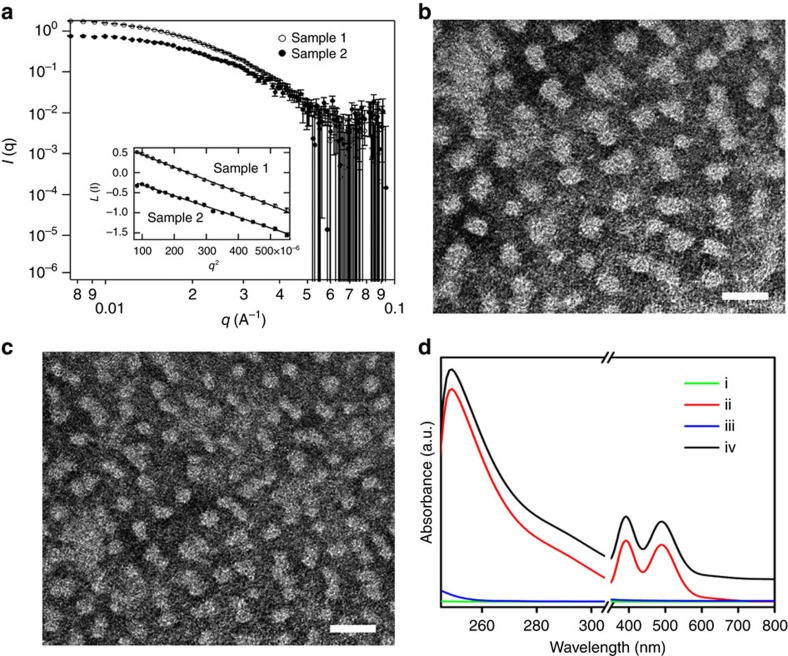
Characterization of polymeric micelles. (**a**) Small-angle neutron scattering (SANS) patterns of two types of polymeric micelle solutions with corresponding Guinier fits in the inset. Sample 1 solution was prepared by mixing 5 mg of PEO-*b*-PMMA, 0.6 ml of DMF, 1 ml of 40 mM Na_3_RhCl_6_ solution in H_2_O, and 1.4 ml D_2_O, while Sample 2 was prepared by mixing 5 mg of PEO-b-PMMA, 0.6 ml of DMF, and 1 ml of 40 mM Na_3_RhCl_6_ solution. (**b**,**c**) Typical TEM micrographs showing the polymeric micelles (**b**) before and (**c**) after the addition of Na_3_RhCl_6_ precursor (Scale bar, 50 nm). The polymeric micelles are stained with 1.0 wt% phosphotungstic acid. (**d**) UV-VIS-NIR spectra of different solutions: (i) DMF+Water, (ii) DMF+Water+Na_3_RhCl_6_, (iii) DMF+Water+PEO-*b*-PMMA and (iv) DMF+Water+PEO-*b*-PMMA+Na_3_RhCl_6_, respectively. [Note: to highlight the interactions among different compositions, UV-VIS-NIR spectra of low- and high- wavelengths were obtained at different composition concentrations: low concentration for low-wavelength, and high concentration for high-wavelength].

Our approach for the synthesis of mesoporous Rh nanoparticles is based on the chemical reduction of Rh precursor in the presence of a polymeric micelle network (Fig. 1). This network functions as a pore-directing agent and template that directs the formation of uniformly sized mesoporous nanoparticles. In pure DMF, the block polymer PEO-*b*-PMMA is completely dissolved as a unimer because of the good solubility of hydrophilic PEO and hydrophobic PMMA segments. Adding deionized water and various aqueous precursor solutions (that is, Na_3_RhCl_6(aq)_ and AA_(aq)_) decreases the solvation of the hydrophobic PMMA segments, leading to the formation of spherical micelles composed of a PMMA core surrounded by a PEO shell. The presence of micelles in solution is clearly illustrated by the Tyndall effect (Supplementary Fig. 5). Small-angle neutron scattering (SANS) was also performed to characterize the micelles in solution (Fig. 3a). The polymeric micelles are relatively monodisperse in solution as displayed by the linear Guinier region at low Q. The radii of gyration of the scattering particles were extracted from the Guinier fits and found to be 100 Å for the sample (Supplementary Fig. 6). Real space radii of gyration extracted from probability distribution functions agree within error to those given by the Guinier fits. In order to directly visualize the micelle structure, polymeric micelles were stained with phosphotungstic acid and further studied by TEM. The bright spheres correspond to the PMMA micellar cores (lower electron density region) due to the negative staining by phosphotungstic acid (Fig. 3b,c and Supplementary Fig. 7). After addition of Na_3_RhCl_6_, no significant changes on the micelle shape were observed from the TEM micrographs, showing the presence of Na_3_RhCl_6_ precursor did not destroy the micelle structure. The average micelle size obtained by SANS (∼20 nm) is larger than the size of PMMA micellar cores in TEM due to micelle shrinkage at high vacuum.

### Applications

**Figure 4:**
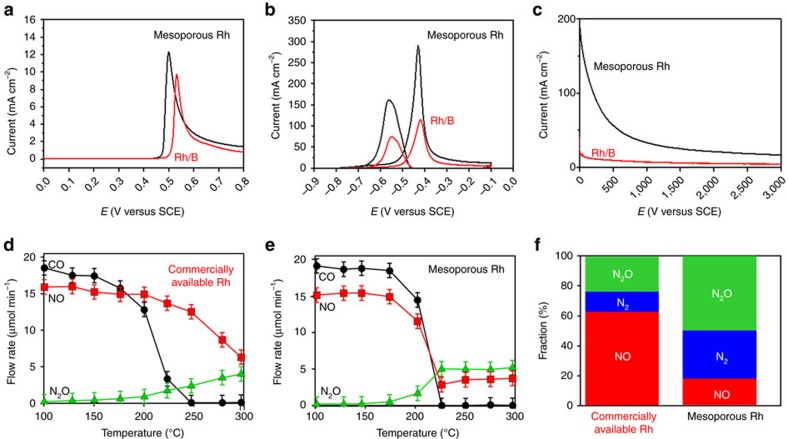
Application of mesoporous Rh nanoparticles. (**a**) Linear sweep voltammograms (LSV) of CO oxidation in 0.5 M H_2_SO_4_ solution and (**b**) CV curves of mesoporous Rh nanoparticles and Rh/B in 1 M KOH+1 M CH_3_OH solution at 50 mV s^−1^. (**c**) Chronoamperometric curves of mesoporous Rh nanoparticles and Rh/B in 1 M KOH+1 M CH_3_OH solution at −0.4 V. (**d**,**e**) The volume fractions of CO, NO and N_2_O in the effluent gas passing through (**d**) the commercially available Al_2_O_3_-supported Rh catalyst (Rh/Al_2_O_3_) and (**e**) the mesoporous Rh nanoparticles at different temperatures. (**f**) Volume fraction of NO (red), N_2_ (blue) and N_2_O gas in the effluent gas at 250 °C.

The catalytic activity for the methanol oxidation reaction (MOR) was investigated electrochemically. The peak current density is conventionally used to evaluate catalytic activity for MOR. In Supplementary Fig. 8, clear features of the hydrogen adsorption/desorption could be observed in the low potential region on both the mesoporous Rh and commercial Rh black (Rh/B) catalysts, indicating the cleanliness of the catalyst surface. For the CO stripping reaction in 0.5 M H_2_SO_4_, linear sweep voltammetry (LSV) was used to oxidize monolayer CO adsorbed on the Rh surface, yielding a characteristic CO oxidation peak on both the mesoporous Rh and Rh/B (Fig. 4a). The mesoporous Rh nanoparticles have a more negative onset potential and peak potential for CO oxidation than Rh/B. This result demonstrates that the mesoporous Rh nanoparticles are better at CO oxidation than Rh/B probably due to higher activity for CO stripping on the curved surface of Rh (Supplementary Fig. 3c,d)^47^,^48^. Figure 4b shows typical cyclic voltammetric curves for MOR catalysed by the two catalysts in 1 M KOH solution containing 1 M methanol at a scan rate of 50 mV s^−1^. The mesoporous Rh nanoparticles exhibited a mass activity of 288 mA mg^−1^ in the forward sweep, which is up to 2.6 times greater than Rh/B catalyst (110 mA mg^−1^), demonstrating the higher utilization efficiency of Rh metal in the mesoporous structures. The chronoamperometric curve (Fig. 4c) recorded at −0.4 V under the same conditions for MOR show that the mesoporous Rh nanoparticles had a slower current decay, because mesoporous structures are known to be less vulnerable to aggregation^49^,^50^.

Generally, structural thermostability is a very important factor for practical catalysts. For reactions that occur at relatively high temperatures, it is critical that the catalysts are able to retain their shape and structure. To study the thermostability of mesoporous Rh, the samples were calcined at different temperatures (for example, 250, 300, 350 and 400 °C) for 1 h in N_2_ or air atmosphere. No obvious changes in the mesoporous structure or particle aggregation could be observed in the SEM micrographs even after thermal treatment at 400 °C in either atmosphere (Supplementary Figs 9 and 10). The low-angle X-ray diffraction profiles are unchanged even after thermal treatment, showing that calcination at high temperatures did not result in the collapse of mesoporous structure (Supplementary Fig. 11). These results indicate that the mesoporous Rh nanoparticles are stable at relatively high temperatures. Supplementary Fig. 12a shows wide-angle X-ray diffraction patterns indicating that the Rh is partially oxidized when the sample was calcined in air. This result was supported by TG-DTA analysis (Supplementary Fig. 12c). However, no oxidized Rh could be observed by X-ray diffraction when the sample was calcined under N_2_ atmosphere (Supplementary Fig. 12b,d). With increasing temperatures, each crystal gradually merged, causing the average size of the Rh nanoparticles to increase slightly from *ca.* 4.2 nm to *ca.* 5.9 nm (for 250 °C), *ca.* 6.2 nm (for 300 °C), *ca.* 6.8 nm (for 350 °C) and *ca.* 7.0 nm (for 400 °C), respectively, under N_2_ atmosphere. After the calcination at 400 °C, the average crystal size was at most *ca.* 7.0 nm which is less than the thickness of the pore walls. Such merging and rearrangement of crystals inside the frameworks did not affect the mesoporous architecture. Heating the structures above 450 °C in N_2_ caused the average crystal size to increase (*ca.* 26.9 nm) beyond the original thickness of the pore walls, destroying the original mesoporous structure (Supplementary Fig. 10e).

Although transition-metal oxides have been investigated for several decades as an alternative material for nitric oxide (NO)-remediation^51^, they are susceptible to rapid degradation by catalytic poisoning by O_2_ contained in the exhaust^52^. We evaluated the performance of the mesoporous Rh and commercially available Al_2_O_3_-supported Rh nanoparticles (2–3 nm Rh nanoparticles) (Rh/Al_2_O_3_; Aldrich, Rh loading=5 wt%) (Supplementary Fig. 13) for the remediation of automobile exhaust under lean-burn conditions (Fig. 4d,e). Each catalyst was subjected to a steady stream of simulated lean-burn exhaust containing nitrogen monoxide (NO), oxygen (O_2_), carbon monoxide (CO) and Ar gas (volume ratio=NO:O_2_:CO:Ar=1:0.5:1:97.5; space velocity=30,000 per 1 h) at different temperatures from room temperature to 300 °C. Figure 4d shows that the CO fraction in the effluent gas decreased with increasing temperature and vanished at 250 °C due to the catalytic oxidation by NO and/or O_2_ over the Rh/Al_2_O_3_. The NO fraction in the effluent gas decreased monotonically with increasing temperature from 15 μmol min^−1^ at 100 °C down to 6 μmol min^−1^ at 300 °C, accompanied by generation of nitrous oxide (N_2_O) via the partial reduction of NO (that is, NO+1/2CO=1/2N_2_O+1/2CO_2_). The decrease in the CO fraction was steeper than the decrease in the NO fraction because CO was preferentially oxidized by O_2_ over the Rh/Al_2_O_3_ catalyst. The effluent gas at 250 °C contained NO, N_2_ and N_2_O, respectively, showing that the Rh/Al_2_O_3_ converted less than 40% of NO into less-toxic N_2_ or N_2_O (Fig. 4f). In contrast, the NO fraction exposed to the mesoporous Rh/Al_2_O_3_ (Supplementary Fig. 14) decreased as steeply as the CO fraction to reach a plateau of 2.5 μmol min^−1^ at 250 °C (Fig. 4e). The N_2_O fraction had an upwards trend as a result of the partial reduction of NO, approaching to 5 μmol min^−1^ at 250 °C and higher temperatures. The effluent gas at 250 °C (Fig. 4f) shows that 85% of the NO gas was converted to N_2_ and/or N_2_O by the mesoporous Rh nanoparticles. It is clear that the mesoporous Rh exhibits superior activity over Rh/Al_2_O_3_ catalyst for the remediation of NO in lean-burn exhaust containing high concentrations of O_2_ (Further discussion is shown in Supplementary Fig. 15.)

## Discussion

The conformational state of the dissolved metal ions in the precursor solution depends sensitively on the interaction between the micelles and metal ions, which impacts the templated formation of mesoporous metal structure^53^,^54^. To understand the conformation state of Rh ions, the precursor solutions containing the PEO-*b*-PMMA micelles and Na_3_RhCl_6_ were studied with ultraviolet-visible (UV-VIS) absorption spectroscopy. The spectra (ii) and (iv) in Fig. 3d display peaks characteristic of Na_3_RhCl_6_ at around 390 nm (^1^A_1g_–^1^T_1g_, spin allowed) and 488 nm (^1^A_1g_–^1^T_2g_, spin allowed) and a weakly observed shoulder at 650 nm (^1^A_1g_–^3^T_1g_, spin forbidden), matching a typical *d*–*d* transition of Rh^3+^ ions. A previous study shows that the [RhCl_6_]^3−^ ion should have corresponding signals at 411, 518 and 680 nm in principle^55^. The UV-VIS absorption spectrum of Na_3_RhCl_6_ (Supplementary Fig. 16) dissolved in pure DMF (in the absence of water) also supports the previous demonstration that [RhCl_6_]^3−^ ions have signals at 411 nm, 518 nm, indicating solvent does not have a huge effect the absorption spectrum of Na_3_RhCl_6_. On the other hand, the UV-VIS absorption spectrum of [Rh(H_2_O)_6_]^3+^ should have corresponding peaks at 311 and 396 nm^55^. This large blueshift is due to water being a *σ*-donor only ligand, whereas Cl^−^ ions are both *σ*- and *π*-donors and weak field ligands. Thus, the peak positions at 390 and 488 nm observed in Fig. 3d are located between the [RhCl_6_]^3−^ ion (411 nm, 518 nm) and the [Rh(H_2_O)_6_]^3+^ (311 nm, 396 nm), indicating there is a partial ligand exchange between H_2_O and Cl^−^ in the coordination sphere of the Rh^3+^ species. This is important because the proposed ligand exchange can modify the charge of the Rh(III) complex in solution (from 3− to 3+ depending on the number of water molecules in the coordination sphere). Since the observed blueshift is small it is likely that the majority of Cl^−^ ions are in the coordination sphere and the complex is negatively charged ([Rh(H_2_O)_3–*x*_Cl_6–*x*_]^(3–*x*)−^).

We hypothesized that the Na_3_RhCl_6_ crystals are dissolved into Na^+^ and [Rh(H_2_O)_3–*x*_Cl_6–*x*_]^(3–*x*)−^ ion complexes. The Rh species interacts with the micelles through hydrogen bonding, ion-dipole interaction as well as water species that are influenced by the ions (anionic and cationic complex species) on the ethylene oxide surface of the micelles. These interactions collectively enhance the concentration of Rh complexes around the micelles and further drive the reaction process. Moreover, we consider that some of the [Rh(H_2_O)_3–*x*_Cl_6–*x*_]^(3–*x*)−^ species exist as free ions in solution as well as in the hydrophilic domains of the PEO-*b*-PMMA micelles. Since the micelles are relatively large, they can support a lot of Rh(III) species within the EO shells. Reduction of Rh species in close vicinity to the micelles, combined with PEO-Rh metal interactions, help assemble and cement the mesostructured Rh nanoparticles.

To directly observe this growth process, intermediate products at different reaction times were investigated with TEM (Supplementary Fig. 17). At the earliest stages of the reaction, tiny Rh nanocrystals were observed in the precursor solution, which were likely stabilized by the EO chains of the micelles. As the reaction proceeded, the tiny crystals grew larger along with the spherical PEO-*b*-PMMA micelles. At this point the mesoporous structure started to appear. After 75 min, the interconnected mesoporous architecture of the Rh nanoparticles began to form close-packed micelles. Beyond this time, no significant change in the shape and pore structure was observed, except that the size of the particles increased. The progress of Rh reduction can also be visually observed via the colour change of the reaction solution, as shown in Supplementary Fig. 18. The solution begins as red solution of molecular precursors that turn brown and then progressively black as the Rh is converted into optically lossy Rh metal. The combination of TEM and optical results supports the formation mechanism based on a chemical reduction process.

We have demonstrated the first synthesis of mesoporous Rh nanoparticles using chemical reduction on a soft template. The formation mechanism indicates that the dissolved metal ions coordinate to the micelle surface and assist in the nucleation and growth of the Rh metal. These mesoporous Rh nanoparticles have high surface area with abundant low-coordination atoms and surprisingly exhibit great thermal stability. As a result, the mesoporous Rh nanoparticles have superior electrocatalytic performance in the MOR compared to commercial Rh catalyst. These nanoparticles also exceed the performance of commercial Rh catalyst for NO remediation of automobile exhaust under lean-burn conditions. We believe that the soft-templating route to mesoporous materials is a robust platform to prepare various kinds of mesoporous metallic nanoparticles and nanostructured films with uniform porous architectures. Our approach applies simple solution chemistry methods that are different in concept from traditional soft-templating and hard-templating approaches. We believe the simplicity and ease of the method will enable better utilization of rare metals in industrially relevant catalytic reactions.

## Methods

### Preparation of mesoporous Rh nanoparticles

The synthesis of mesoporous Rh nanoparticles is based on a wet chemical reduction process. In a typical synthesis, 5 mg of poly(ethylene oxide)-*b*-poly(methyl methacrylate) (PEO_(10500)_-*b*-PMMA_(18000)_) was completely dissolved in 0.6 ml N,N-DMF. Then, 0.4 ml of deionized water, 1 ml of aqueous 40 mM Na_3_RhCl_6_, and 1 ml of aqueous 100 mM AA were added to the above DMF solution in sequence, resulting in a transparent light-brown coloured solution. The reaction solution was kept in water bath for 12 h at 60 °C. After the solution colour changed from light-brown to completely black, the samples were collected by centrifugation at 14,000 r.p.m. for 20 min and the residual PEO_(10500)_-*b*-PMMA_(18000)_ was removed by five consecutive washing/centrifugation cycles with acetone and water and used for characterization.

### Characterization

Field emission scanning electron microscope (SEM, HITACHI SU-8000) was used to observe morphology of mesoporous Rh nanoparticles with the accelerating voltage of 5 kV. High-resolution transmission electron microscopy (HRTEM, JEOL JEM-2100F) operated at 200 kV was used to investigate the interior structure of the mesoporous nanoparticles. The samples for TEM and HRTEM measurements were prepared by depositing a drop of the diluted colloidal suspension on a TEM grid. Powder X-ray diffraction measurements were conducted on a Smart lab X-ray diffractometer (RIGAKU) at a scanning rate of 1 deg min^−1^ with a Cu Kα radiation (40 kV, 30 mA) source. Small-angle X-ray scattering (SAXS) measurements (Rigaku NANO-Viewer) were used to evaluate the pore-to-pore distance. The SAXS instrument used a Cu Kα radiation (40 kV, 30 mA) source with a camera length at 700 mm. SANS measurements were performed at the Australian Nuclear Science and Technology Organisation on the Quokka instrument. Neutrons of 5 Å wavelength were used with sample to detector distances of 8 and 1.3 m. Samples were corrected for background measurements and placed on an absolute scale using standard procedures.

### Electrochemical measurement

Electrochemical investigations were performed using a CHI 842B electrochemical analyzer (CHI Instrument, USA) to perform cyclic voltammograms, and chronoamperometric curves (CA) for mesoporous Rh nanoparticles and commercially available Rh black (Rh/B). The three-electrode cell consists of a reference electrode (saturated calomel electrode, SCE), a counter electrode (Pt wire) and a working electrode (glassy carbon electrode, GCE). The modified GCE was coated with the mesoporous Rh sample (5.0 μg) and dried at room temperature. Then, 5.0 μl of Nafion (0.05 wt%) was coated on the surface of the modified GCE and dried before electrochemical experiments. Prior to CO stripping measurements, the GCE modified with the mesoporous Rh sample was electrochemically activated by potential cycling between −0.4 V and +0.8 V (versus SCE) in 0.5 M H_2_SO_4_ until the obtained CVs became characteristic of a clean Rh electrode. After that, 0.5 M H_2_SO_4_ solution with GCE was purged by CO gas for 20 min, then was purged by using N_2_ gas for 20 min to removal of extra CO gas in solution. Methanol electro-oxidation measurements were performed in a solution of 1 M KOH solution containing 1 M methanol at a scan rate of 50 mV s^−1^.

### NO remediation measurement

Alumina-supported Rh nanoparticles (Rh/Al_2_O_3_, Aldrich, Rh loading=5 wt%) were used as the control. An aliquot of 5 mg of the mesoporous Rh nanoparticles was mixed with 95 mg of Al_2_O_3_ powder to adjust the apparent Rh loading to that of the control catalyst. Each of the prepared mesoporous Rh/Al_2_O_3_ mixture and the control Rh/Al_2_O_3_ was subjected to a steady flow of simulated exhaust containing equimolar amounts of NO, oxygen (O_2_), carbon monoxide (CO) and argon (Ar) gas (volume ratio=NO:O_2_:CO:Ar=1:0.5:1:97.5; space velocity=30,000 per 1 h) at different temperatures from room temperature to 300 °C. The chemical composition of the effluent gas was analysed with a Fourier transform infrared spectrometer (FTIR, Shimadzu Prestige 21) equipped with a gas cell.

### Data availability

All relevant data that support this study are available within the paper and its Supplementary Information file, or from the corresponding authors upon request.

## Additional information

**How to cite this article:** Jiang, B. *et al*. Mesoporous metallic rhodium nanoparticles. *Nat. Commun.* **8**, 15581 doi: 10.1038/ncomms15581 (2017).

**Publisher's note:** Springer Nature remains neutral with regard to jurisdictional claims in published maps and institutional affiliations.

## Supplementary Material

Supplementary Information Supplementary figures, supplementary table, supplementary notes and supplementary references.
