# Supplementary material for: Mesoporous metallic rhodium nanoparticles
Source: Nat Commun. 2017 May 19;8:15581. doi: 10.1038/ncomms15581 (PMC5454530; doi:10.1038/ncomms15581)
Supplement: Supplementary Information — Supplementary figures, supplementary table, supplementary notes and supplementary references. [file ncomms15581-s1.pdf]

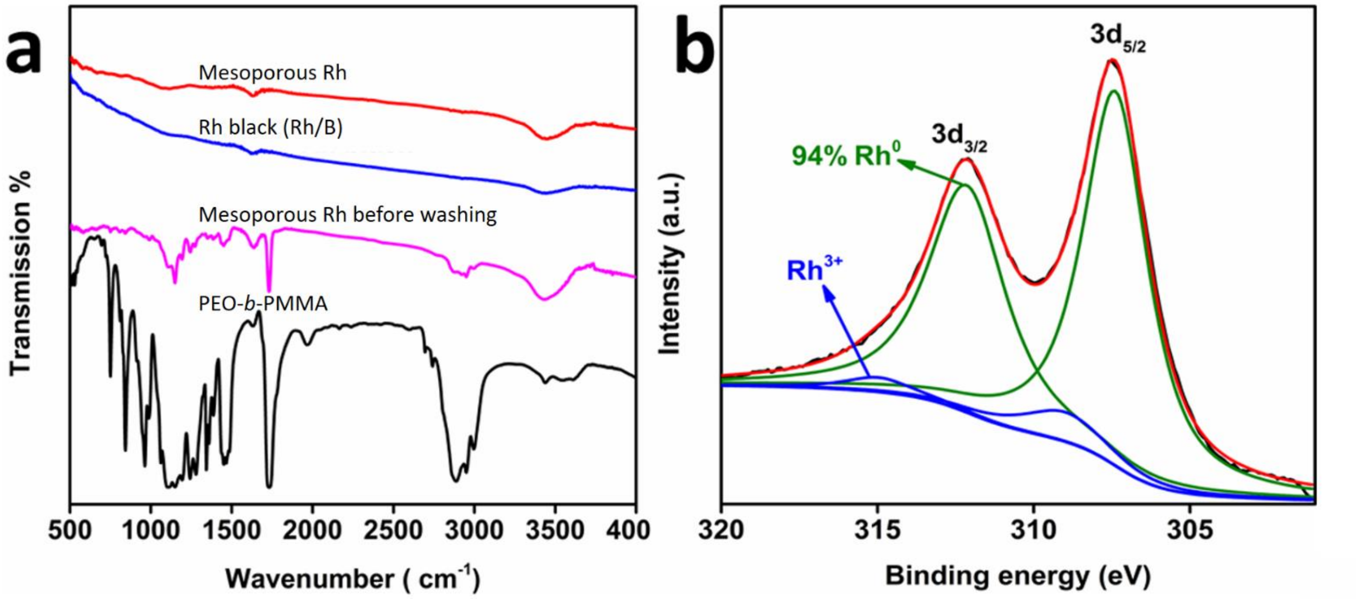

Supplementary Figure 1. FI-IR spectra and XPS spectrum. (**a**) FT-IR spectra of the mesoporous Rh nanoparticles before and after the washing procedure. For reference, additional spectra showing commercially available Rh black (Rh/B) and block polymer PEO-*b*-PMMA, respectively. (**b**) XPS spectrum of the mesoporous Rh nanoparticles after the washing procedure.

Supplementary Note 1: The IR peaks derived from PEO-*b*-PMMA template disappered after the washing procedure. From CHNSO analysis, the carbon and oxygen contents are < 1-2 wt%, respectively. From the XPS data, most of Rh atoms on the surface are not oxidized in air.

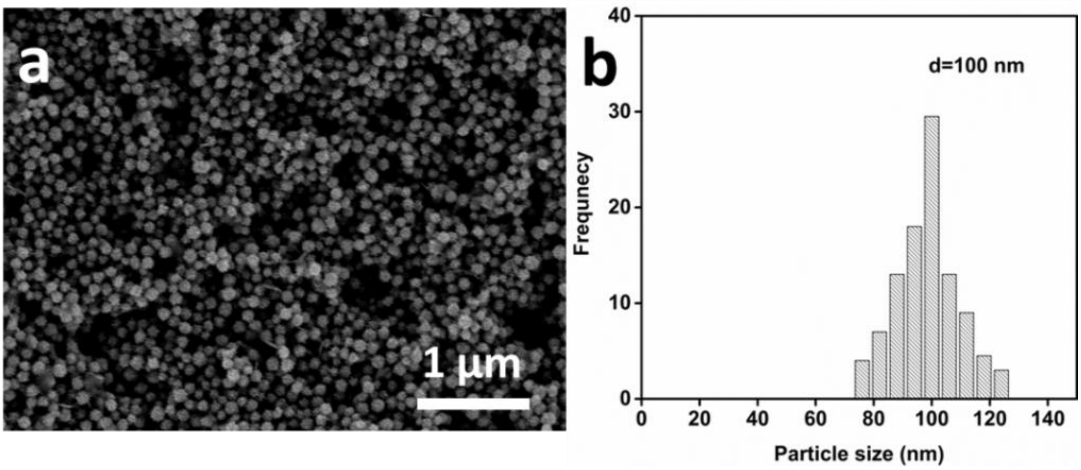

Supplementary Figure 2. SEM micrograph and histogram showing the particle size distribution. (**a**) SEM micrograph of the mesoporous Rh nanoparticles at low magnifications. (**b**) A histogram showing the particle size distribution of the mesoporous Rh nanoparticles.

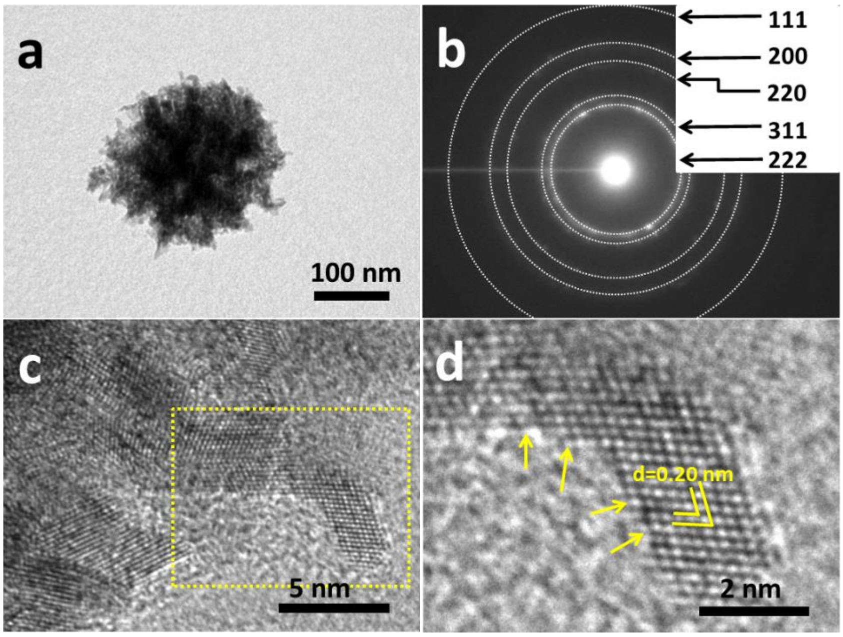

Supplementary Figure 3. TEM study. (**a**) TEM image of a single mesoporous Rh nanoparticle, (**b**) the SAED pattern obtained from the single particle, and (**c, d**) HRTEM images of mesoporous Rh nanoparticles. The kink and step sites are indicated by the yellow arrows.

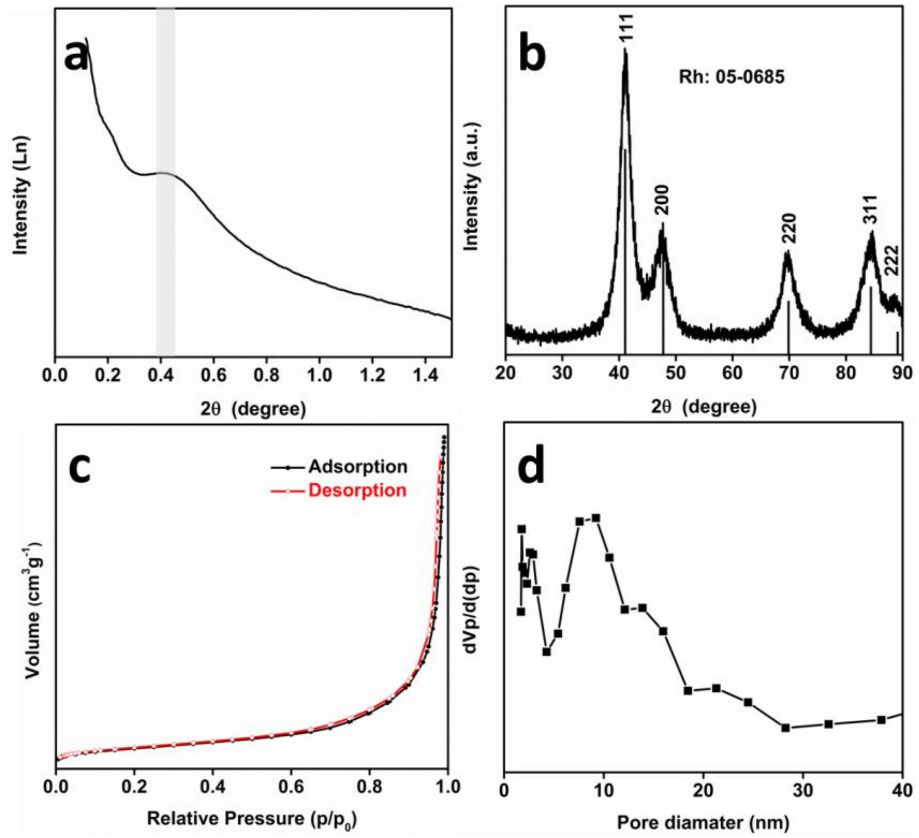

Supplementary Figure 4. Low-, and wide-angle XRD patterns, N_2 adsorption-desorption isotherms, and the BJH pore size distribution curve. (**a**) Low-, and (**b**) wide-angle XRD patterns of mesoporous Rh nanoparticles. (**c**) N_2 adsorption-desorption isotherms and (**d**) the BJH pore size distribution curve of mesoporous Rh nanoparticles.

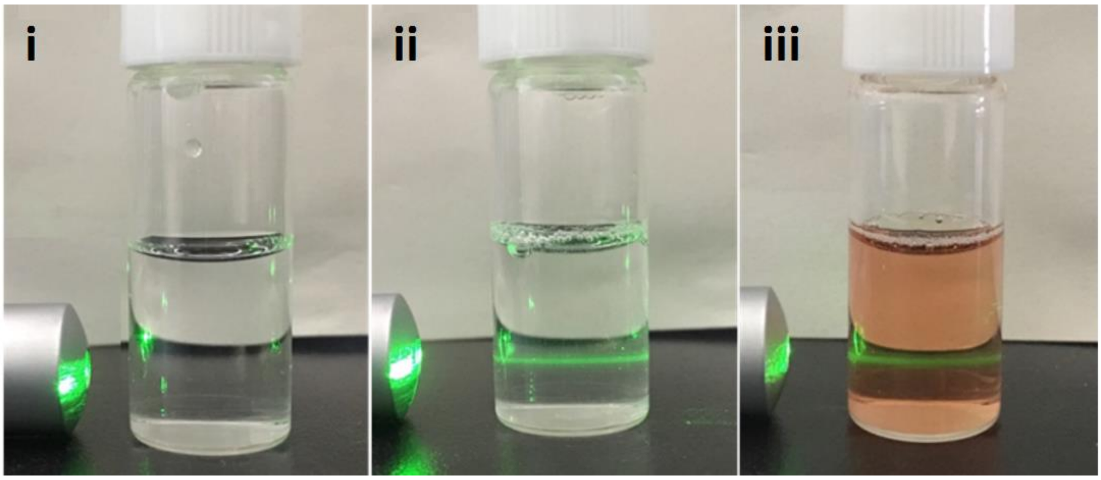

Supplementary Figure 5. Optical micrographs. Optical micrographs demonstrating the emergence of the Tyndall effect in the electrolyte solution with different composition: (i) PEO-*b*-PMMA dissolved in DMF, (ii) PEO-*b*-PMMA dissolved in DMF+water, and (iii) PEO-*b*-PMMA dissolved in DMF+water+Na_3RhCl_6.

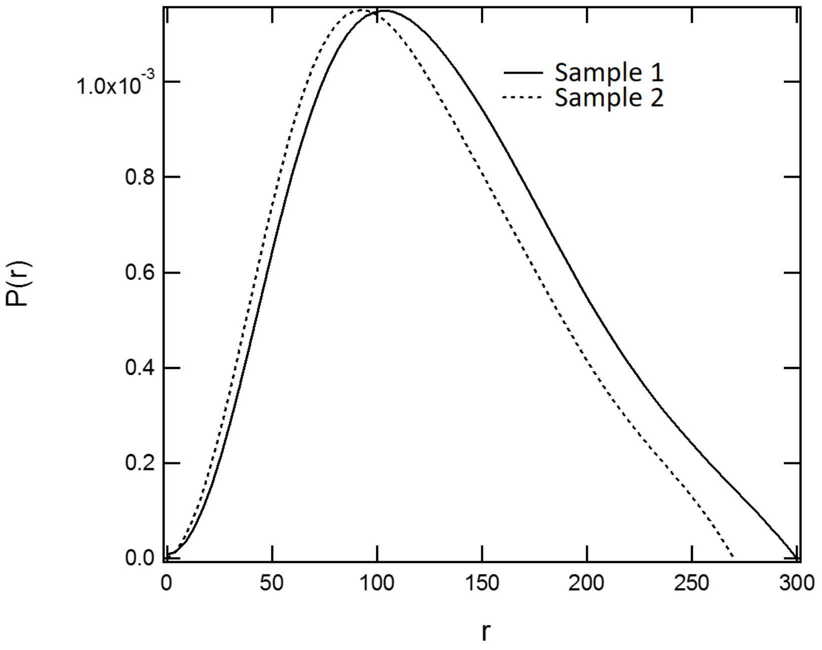

Supplementary Figure 6. Radial probability distribution function extracted from the small angle neutron scattering data. Sample 1 solution was prepared by mixing 5 mg of PEO-*b*-PMMA, 0.6 mL of DMF, 1 mL of 40 mM Na_3RhCl_6 solution in H_2O, and 1.4 mL D_2O, while Sample 2 was prepared by mixing 5 mg of PEO-*b*-PMMA, 0.6 mL of DMF, and 1 mL of 40 mM Na_3RhCl_6 solution.

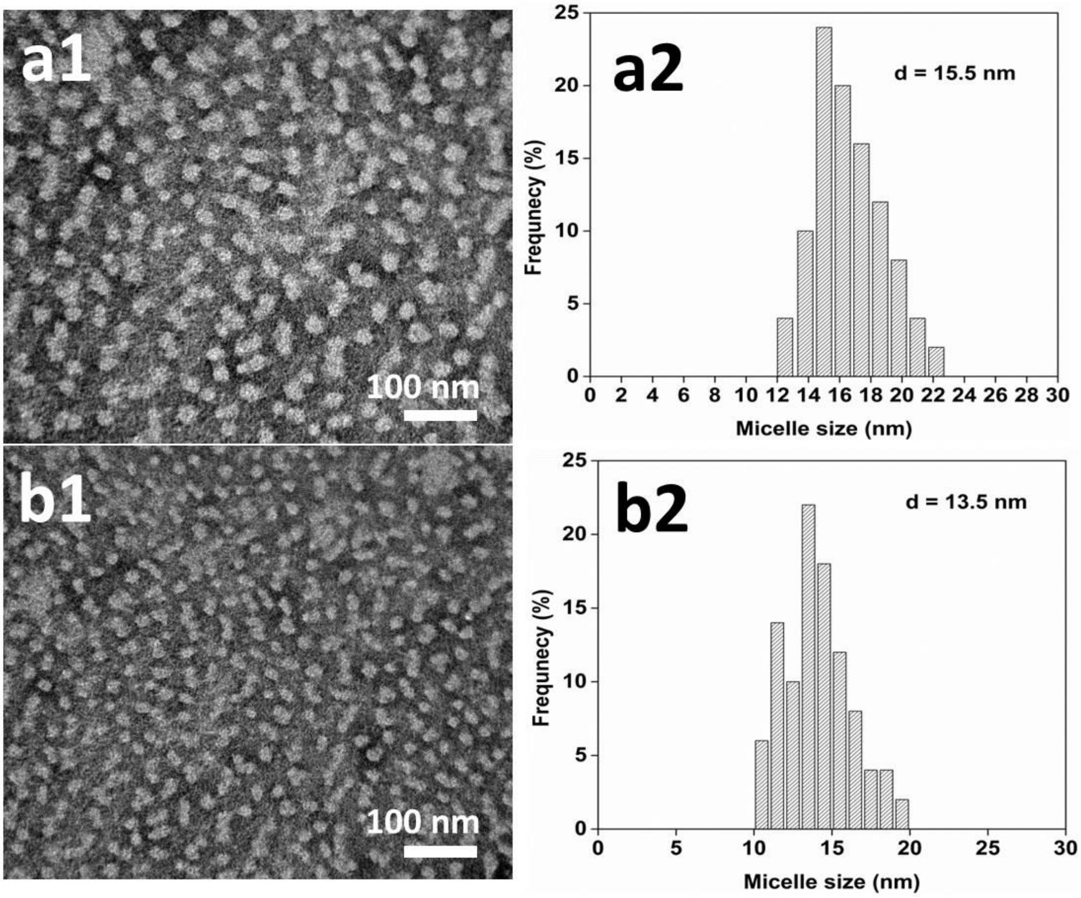

Supplementary Figure 7. SEM micrograph and histogram showing the micelle size distribution. Low-magnification TEM micrographs and histogram showing the size distribution of the polymeric micelles (a) before and (b) after the addition of Na_3RhCl_6. To highlight the micelle structures, the samples were stained with 1.0 wt% phosphotungstic acid.

Supplementary Note 2: Previous report has described the effect of Cl^-, Br^-, and I^- on micellization and the subsequent effect on mesoporous silica (*Langmuir*, **2011**, 27, 7121-7131)). In this series, Cl^- is a hydrotropic ion and that enhances micellization, while the I^- ion is lyotropic and inhibits micellization. The role of hydrotropic anions is structural and somehow effects the aqueous assembly of surfactants into micelles with a smaller corona. The TEM micrographs in **Supplementary Figure 7** also support this conclusion that the micelles become quantitatively smaller upon addition of the Rh salt into the media. It is likely that either $[RhCl_{6-x}(H_2O)]^{(3-x)-}$ precursor or Cl^- ions released into solution due to ligand exchange, enhance micellization. This results in increasingly compact micelles during the further assembly process.

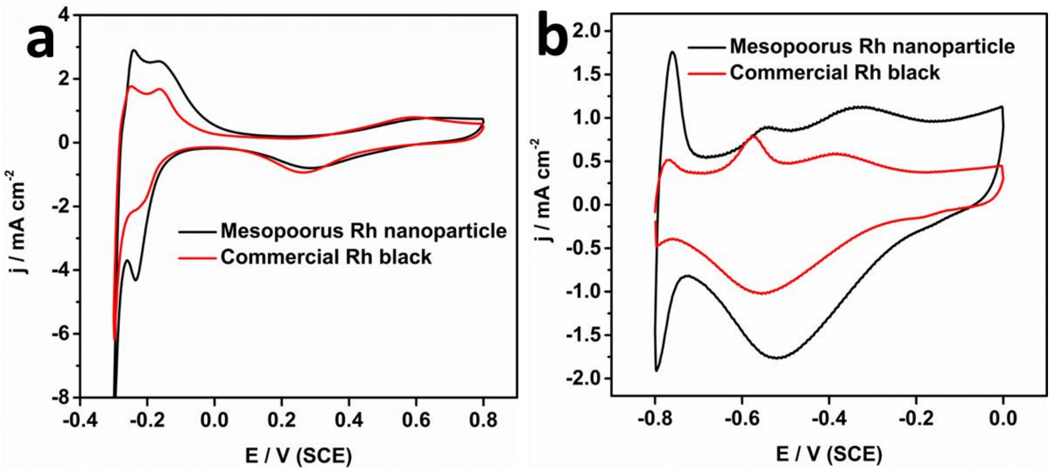

Supplementary Figure 8. Cyclic voltammogram (CV) curves. Cyclic voltammogram (CV) curves of the mesoporous Rh and commercial Rh black in N_2-purged (a) 0.5 M H_2SO_4 solution, and (b) 1 M KOH at 50 $mV{\cdot}s^{-1}$.

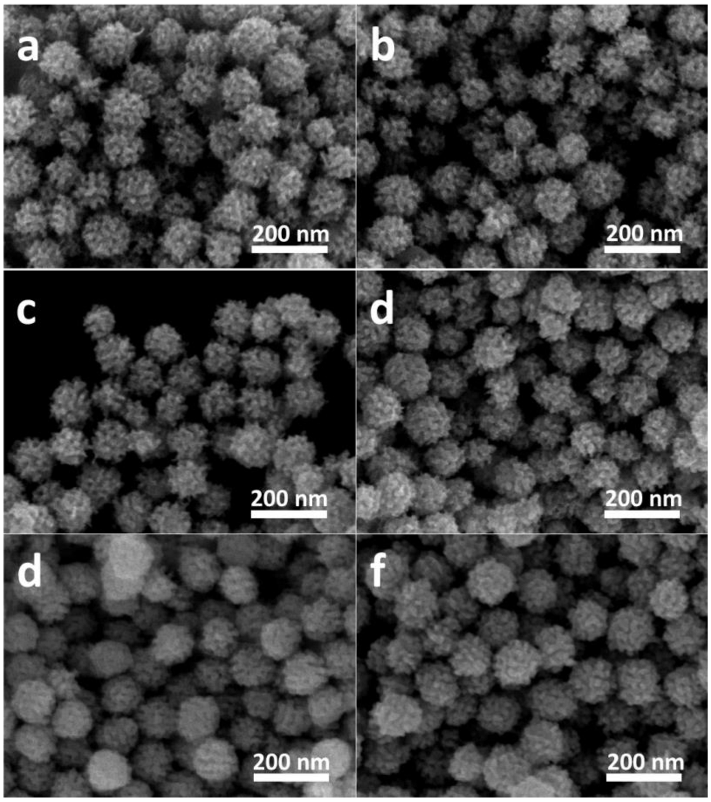

Supplementary Figure 9. SEM micrographs. SEM micrographs of mesoporous Rh nanoparticles after thermal treatment at different temperatures: (a) 250 °C, (b) 300 °C, (c) 350 °C, (d) 400 °C, (e) 450 °C and (f) 500 °C in air atmosphere.

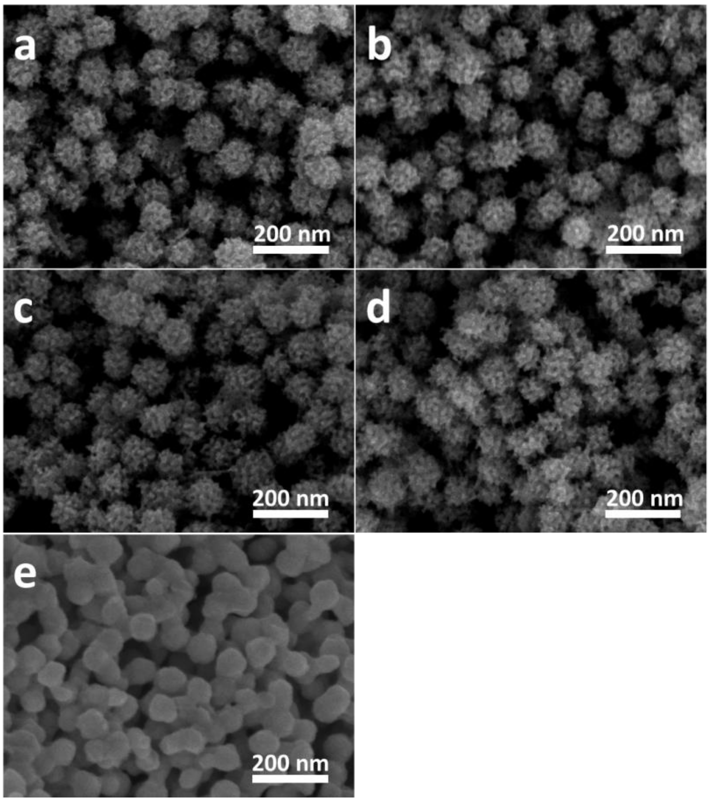

Supplementary Figure 10. SEM micrographs. SEM micrographs of mesoporous Rh nanoparticles after thermal treatment at different temperatures: (a) 250 ^{o}C, (b) 300 ^{o}C, (c) 350 ^{o}C, (d) 400 ^{o}C, and (e) 450 ^{o}C in nitrogen atmosphere.

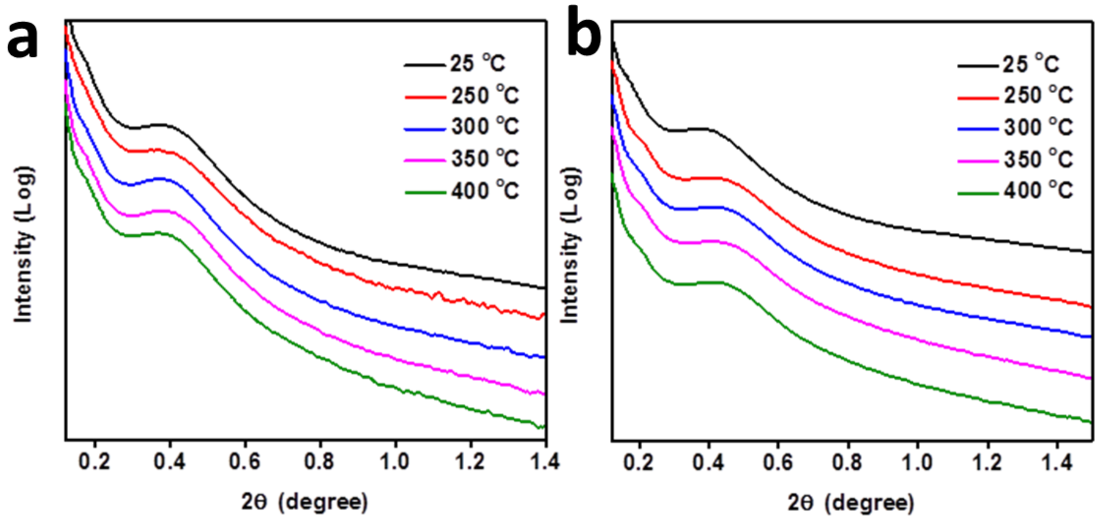

Supplementary Figure 11. Low-angle XRD patterns. Low-angle XRD patterns of mesoporous Rh nanoparticles after thermal treatment at different temperatures (25 °C, 250 °C, 300 °C, 350 °C, and 400 °C) in (a) air atmosphere and (b) nitrogen atmosphere.

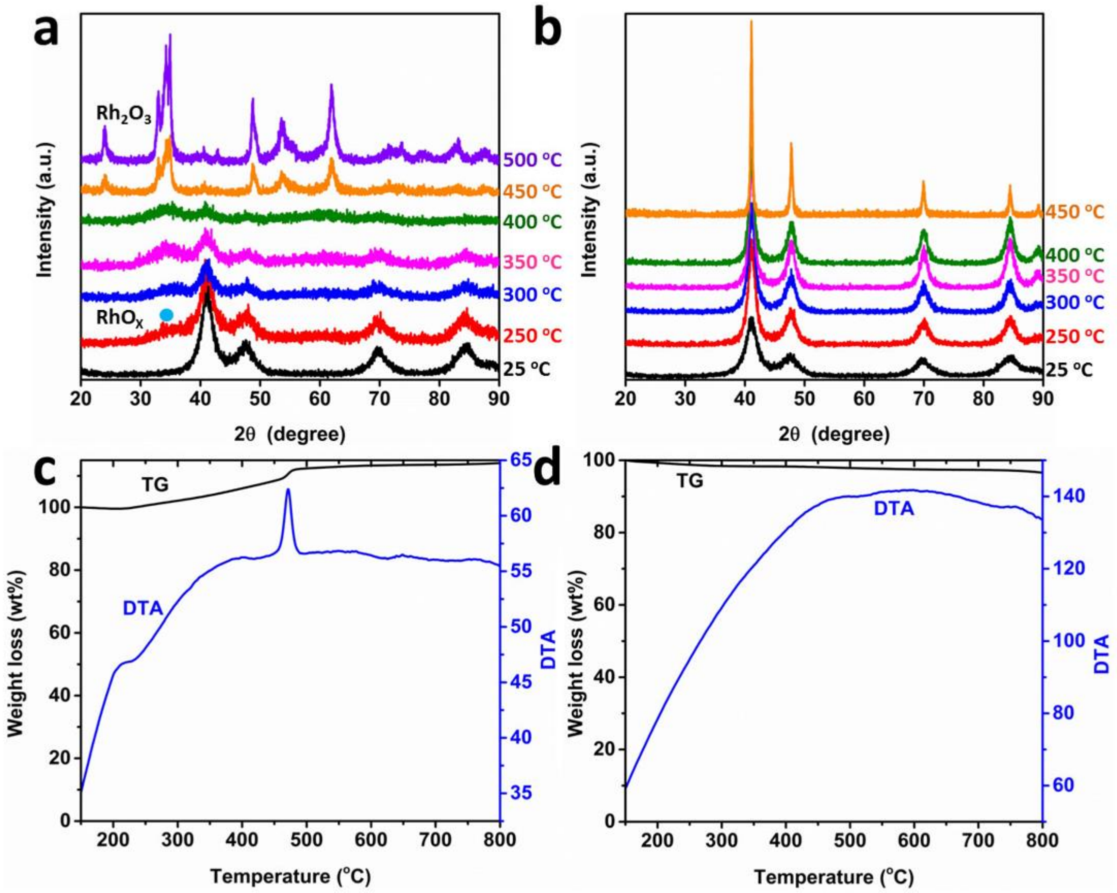

Supplementary Figure 12. Wide-angle XRD patterns and TG/DTA curves. Wide-angle XRD patterns of mesoporous Rh nanoparticles after thermal treatment at different temperatures in (a) air atmosphere (25 °C, 250 °C, 300 °C, 350 °C, 400 °C, 450 °C, and 500 °C) and (b) nitrogen atmosphere (25 °C, 250 °C, 300 °C, 350 °C, 400 °C, and 450 °C). (c) TG/DTA curves measured in air and (d) TG/DTA curves measured in nitrogen for as-prepared mesoporous Rh nanoparticels. The exothermic peak in panel (c) indicates that Rh is oxidizing somewhat.

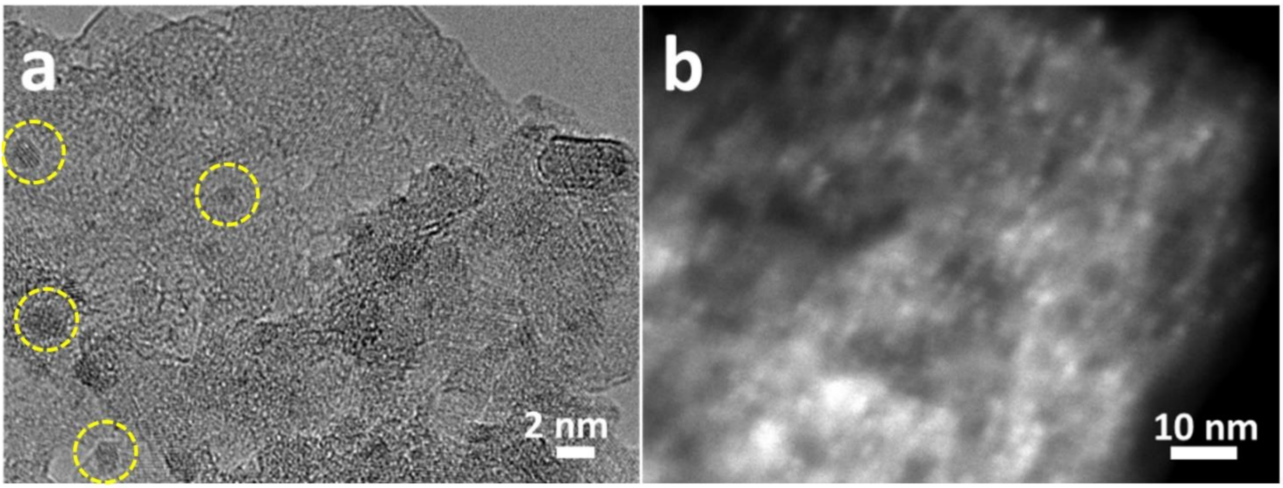

Supplementary Figure 13. TEM study. TEM and HAADF-STEM micrographs showing commercially available Rh/Al_2O_3. Some of the Rh nanoparticles are highlighted with yellow circles.

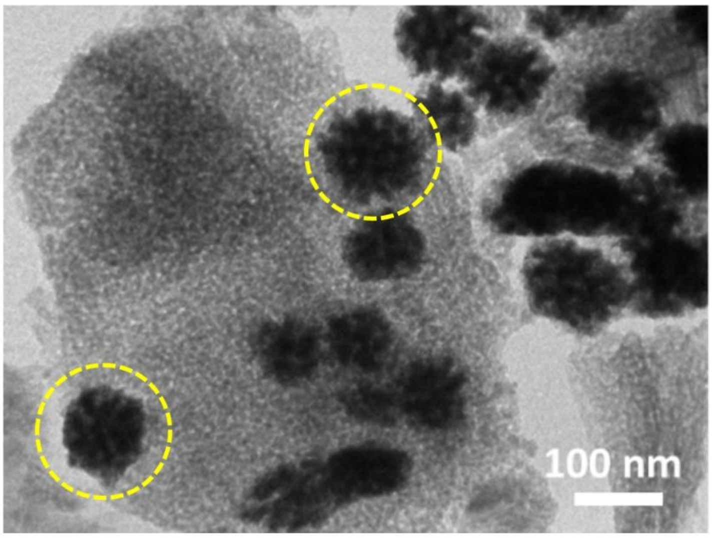

Supplementary Figure 14. TEM study. TEM micrograph of mesoporous Rh/Al_2O_3. Some mesoporous Rh nanoparticles are highlighted with yellow circles.

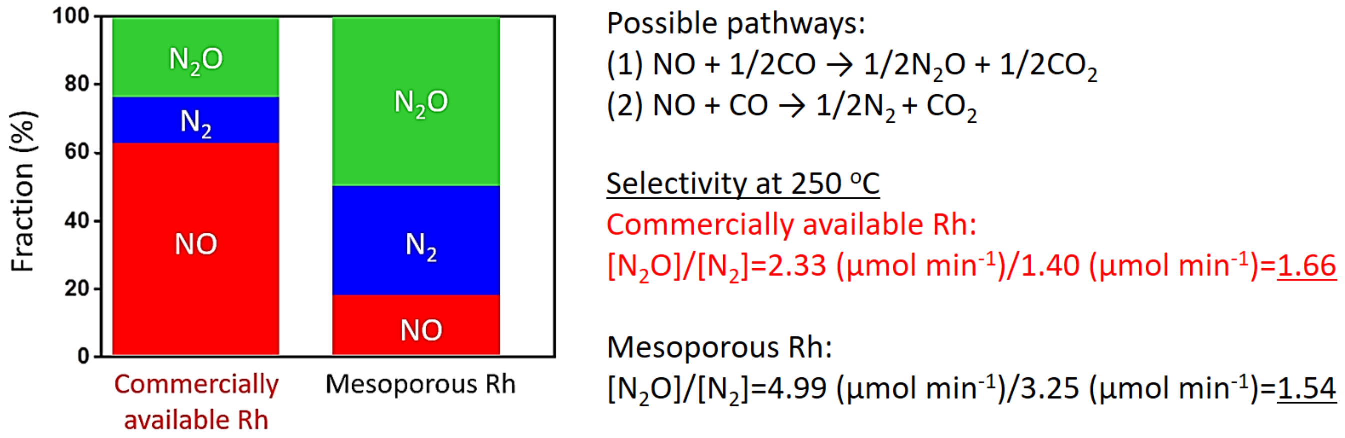

Supplementary Figure 15. Comparison of the calculated selectivity. Comparison of the calculated selectivity of mesoporous Rh nanoparticles with commercially available Rh catalysts.

Supplementary Note 3: The effluent gas flowing over the mesoporous Rh contains 4.99 µmol min^{-1} of N_2O and 3.25 µmol min^{-1} of N_2 at 250 ^{o}C, which are generated via different two reaction pathways. The selectivity for the N_2O generation on mesoporous Rh catalyst is calculated as $[N_2O]/[N_2]$ = 4.99 (µmol min^{-1})/3.25 (µmol min^{-1}) = 1.54. The alumina-supported Rh catalyst (commercially available Rh) also converted NO to N_2O and/or N_2, in which the selectivity for N_2O is calculated as $[N_2O]/[N_2]$ = 2.33 (µmol min^{-1})/1.40 (µmol min^{-1}) = 1.66. This simple calculation shows that the commercially available Rh catalyst has similar selectivity for N_2O as the mesoporous Rh catalyst. However, the mesoporous Rh converts NO into N_2O and/or N_2 much more efficiently with almost the same selectivity for N_2O, resulting in apparently higher N_2O generation. We propose that the mesoporous Rh exhibit high performance due to a combination of high surface area and presence of numerous atomic steps and kink sites which expose unsaturated coordination atoms.

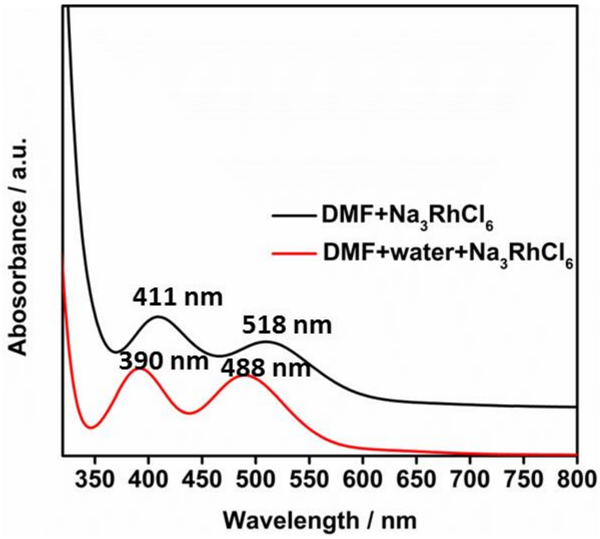

Supplementary Figure 16. UV-Vis-NIR spectra. UV-Vis-NIR spectra of DMF+Na_3RhCl_6 and DMF+water+Na_3RhCl_6, respectively.

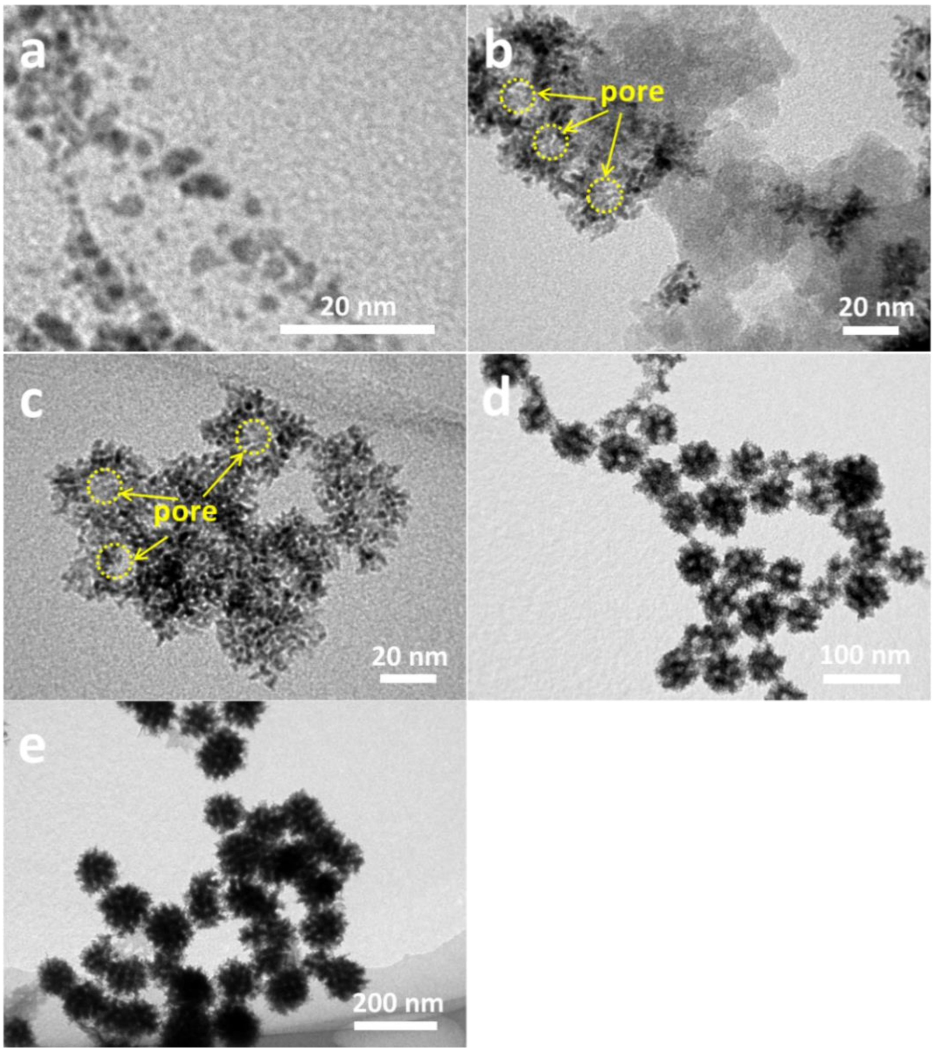

Supplementary Figure 17. TEM micrographs. TEM micrographs of Rh samples taken from different reaction periods: (a) 50 min, (b) 60 min, (c) 75 min, (d) 90 min, and (e) 180 min, respectively.

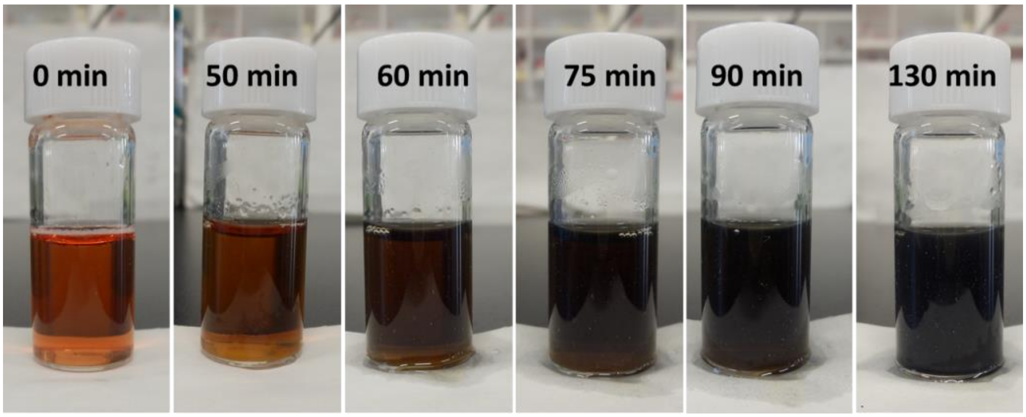

Supplementary Figure 18. Photographs of colloidal suspensions. Photographs of colloidal suspensions of reaction solution taken at different reaction times. The reaction was performed at 60 ^{o}C.

Supplementary Table 1 Summary on surface areas of mesoporous nanoparticles with various compositions.

Sample names	Density (g/cm^3)	Surface area (g/m^2)	Reference
Mesoporous Rh nanoparticles	12.41	50	Present work
Mesoporous SiO_2 nanoparticles	2.65	193-500	1
Mesoporous TiO_2 nanoparticles	4.23	148	2
Ordered porous TiO_2	4.23	135	3
Mesoporous In_2O_3 nanoparticles	7.18	48	4
Mesoporous carbon nanoparticles	1.80-2.10	343	5
Mesoporous SnO_2	6.95	87	6

Supplementary references

[1] Wei, J., Wang, H., Deng, Y., Sun, Z., Shi, L., Tu, B., Luqman, M. Zhao, D. Solvent evaporation induced aggregating assembly approach to three-dimensional ordered mesoporous silica with ultralarge accessible mesopores. *J. Am. Chem. Soc.* **133**, 20369-20377 (2011).

[2] Liu, Y., Lan, K., Li, S., Liu, Y., Kong, B., Wang, G., Zhang, P., Wang, R., He, H., Ling, Y., Al-Enizi, A. M., Elzatahry, A. A., Cao, Y., Chen, G., Zhao D. Constructing three-dimensional mesoporous bouquet-posy-like TiO_2 superstructures with radially oriented mesochannels and single-crystal walls. *J. Am. Chem. Soc.* **139**, 517-526 (2017).

[3] Sun, W., Zhou, S., You, B., Wu L. Facile fabrication and high photoelectric properties of hierarchically ordered porous TiO_2. *Chem. Mater.* **24,** 3800-3810 (2012).

[4] Ren, Y., Zhou, X., Luo, W., Xu, P., Zhu, Y., Li, X., Cheng, X., Deng, Y., Zhao D. Amphiphilic block copolymer templated synthesis of mesoporous indium oxides with nanosheet-assembled pore walls. *Chem. Mater.* **28**, 7997-8005 (2016).

[5] Tang, J., Liu, J., Li, C., Li, Y., Tade, M. O., Dai, S., Yamauchi, Y. Synthesis of nitrogen-doped mesoporous carbon spheres with extra-large pores through assembly of diblock copolymer micelles. *Angew. Chem. Int. Ed.* **54**, 588-593 (2015).

[6] Wang, X., Li, Z., Li, Q., Wang, C., Chen, A., Zhang, Z., Fan, R., Yin, L. Ordered mesoporous SnO_2 with a highly crystalline state as an anode material for lithium ion batteries with enhanced electrochemical performance. *CrystEngComm* **15**, 3696-3704 (2013).
